# Predictors of Outpatient and Inpatient Service Utilization Among Publicly–Insured Youth With Eating Disorders

**DOI:** 10.1002/eat.24301

**Published:** 2024-10-24

**Authors:** Megan E. Mikhail, Kate Duggento Cordell, Amanda E. Downey, Lonnie R. Snowden, Erin C. Accurso

**Affiliations:** ^1^ Department of Psychiatry and Behavioral Sciences University of California San Francisco California USA; ^2^ Michigan State University East Lansing Michigan USA; ^3^ Mental Health Data Alliance Folsom California USA; ^4^ Center for Innovation in Population Health University of Kentucky Lexington Kentucky USA; ^5^ Social Policy Institute, San Diego State University San Diego California USA; ^6^ Department of Pediatrics University of California, San Francisco San Francisco California USA; ^7^ School of Public Health University of California Berkeley California USA; ^8^ Philip R. Lee Institute for Health Policy Studies, School of Medicine University of California San Francisco California USA

**Keywords:** anorexia nervosa, bulimia nervosa, disadvantage, eating disorders, health disparities, Medicaid, other specified feeding or eating disorders, treatment, youth

## Abstract

**Introduction:**

Although eating disorders (EDs) affect youth from all socioeconomic backgrounds, little is known about the treatment experiences of under‐resourced youth with EDs. To address this gap, we examined patterns of outpatient and inpatient service utilization among publicly–insured youth with EDs in California and potential disparities for youth with additional marginalized identities.

**Method:**

Participants were identified from the full sample of California Medicaid/Medi‐Cal beneficiaries aged 7–18 with ≥ 1 service episode between January 1, 2014 and December 31, 2016. Claims data were extracted for youth with a full year of claims after the first known ED diagnosis (*N* = 3311) to analyze outpatient mental health, outpatient medical/physical, inpatient mental health, and inpatient medical/physical service use across ED diagnosis and demographic characteristics (sex, age, race/ethnicity, and preferred language).

**Results:**

Outpatient individual and family therapy service utilization was low across ED diagnoses (4–7 individual therapy sessions and ≈5 family therapy sessions annually). Conversely, hospitalization rates were high, particularly among youth with anorexia nervosa (27.8%) and bulimia nervosa (30.0%). Youth with other specified feeding or ED had high medical service utilization, with more days of outpatient medical care and greater odds of medical hospitalization than youth with all other diagnoses. Latinx youth, Black youth, and boys tended to receive fewer services after accounting for diagnosis, with disparities particularly pronounced for Latinx youth.

**Conclusions:**

Publicly–insured youth with EDs in California experience high hospitalization rates but receive limited outpatient therapy. Additional research is needed to identify possible unmet needs and factors contributing to treatment disparities among these youth.

**Summary**

- Publicly–insured youth with eating disorders (EDs) in California experienced unfavorable service use patterns: high hospitalization rates but low utilization of individual and family therapy.
- After accounting for ED diagnosis, Latinx youth received fewer mental health and medical services across levels of care.
- Results suggest potential unmet treatment needs in under‐resourced youth with EDs, particularly those with multiple marginalized identities.

Eating disorders (EDs, e.g., anorexia nervosa [AN], bulimia nervosa [BN], binge‐eating disorder [BED], and other specified feeding or ED [OSFED]) have been stereotyped as predominantly impacting White, affluent youth (Gard and Freeman 1996; Sonneville and Lipson 2018). Population‐based studies suggest little empirical basis for this stereotype, with equal or greater rates of EDs among people of color (Mikhail and Klump 2021; Rodgers, Berry, and Franko 2018; Swanson et al. 2011) and youth experiencing socioeconomic disadvantage (Carroll et al. 2023; Huryk, Drury, and Loeb 2021; Mikhail et al. 2021, 2023). Nevertheless, the widespread perception of EDs as “diseases of affluence” has contributed to significant disparities in detection (Sim et al. 2024; Becker et al. 2003) and treatment (Marques et al. 2011; Moreno et al. 2023). Minoritized populations are also underrepresented in ED research (Egbert et al. 2022; Halbeisen, Brandt, and Paslakis 2022; Mikhail and Klump 2021), hindering understanding of their current treatment experiences and potential unmet needs (e.g., access to evidence‐based care). More studies are therefore urgently needed to facilitate timely, effective intervention for youth with EDs from marginalized and under‐resourced backgrounds.

California's public Medicaid insurance program (Medi‐Cal) offers a unique context for understanding the treatment experiences of under‐resourced youth with EDs. Medi‐Cal is California's largest healthcare purchaser and provides services for approximately one‐third of the state (California Department of Health Care Services [DHCS] 2024a). Over 5.5 million youth in California receive healthcare coverage through Medi‐Cal, approximately 88% of whom are youth of color (including ~60% who identify as Hispanic/Latinx) and a third of whom speak a primary language other than English (DHCS 2024b). Medi‐Cal is a decentralized program independently administered by California's 58 counties, which span diverse service environments that differ substantially in size, urbanicity, socioeconomic context, and provider capacity. Thus, Medi‐Cal captures a wide range of healthcare experiences despite representing a single state.

Our group recently published the first report characterizing publicly–insured youth with EDs in the United States (US) using Medi‐Cal claims data (Accurso et al. 2024). Youth with EDs in this sample were diverse in terms of race and ethnicity (80% youth of color, 59% Latinx), sex (30% male), and preferred language (49% Spanish). Interestingly, youth with EDs were even more diverse on several dimensions than their counterparts with other mental health conditions (e.g., more likely to be Latinx and Spanish speaking than youth with mood/anxiety or psychotic disorders). This study also began to capture some of the potential disparities experienced by this population. For example, over half of youth with EDs had an unspecified feeding or ED (UFED) diagnosis, which could suggest insufficient time or availability of providers with specialized ED training to make a more specific diagnosis. Since diagnosis informs treatment, this lack of diagnostic clarity could impede the delivery of appropriate interventions.

Available evidence suggests public healthcare systems are not well‐equipped to care for youth with EDs (Accurso, Buckelew, and Snowden 2021; Crest et al. 2024), but little is known about the specific treatment experiences of publicly–insured youth with EDs in the United States. Understanding patterns of service utilization is important to contextualize current treatment experiences and identify potential gaps in care for youth from under‐resourced backgrounds. It may also help illuminate treatment inequities for publicly–insured youth with other marginalized identities (e.g., youth of color). Finally, examining service utilization by diagnostic category can provide insight into treatment needs and disparities across ED diagnoses, including whether lack of diagnostic specificity has treatment implications.

In the current study, we examined patterns of mental and medical/physical health service utilization among Medi‐Cal‐insured youth with EDs, including the extent to which service use differed across diagnostic categories and key demographic variables (age, sex, racial/ethnic identity, and interactions between these factors). We also performed targeted analyses to better understand the characteristics of youth who were hospitalized within the first year after a known ED diagnosis given the severity and high costs that hospitalization denotes (Weissman and Rosselli 2017). To our knowledge, this is the first study detailing service utilization across diagnostic and demographic characteristics among publicly–insured youth with EDs in the US, which is a critical step to inform care needs.

## 1 Method

### 1.1 Participants

Participants were drawn from the full sample of Medi‐Cal beneficiaries aged 7–18 with at least one service episode between January 1, 2014, and December 31, 2016 (*N* = 4,819,221 unique beneficiaries). Current analyses involved the 8075 youth who received a primary or secondary ED diagnosis at any point in that period. The demographics of this sample have been described in detail elsewhere (Accurso et al. 2024).

Youth commonly move in and out of the Medi‐Cal system as their family's financial and other circumstances change. To ensure service utilization was measured over the same timeframe for all youth, primary analyses focused on youth with EDs who were continuously enrolled in Medi‐Cal with a full year of claims after their first known diagnosis (*n* = 3311). Descriptive statistics are also provided for youth with a full second year of claims after the first known diagnosis (*n* = 1293). EDs were classified into exclusive diagnostic groups using the last available ICD‐9 or ICD‐10 diagnosis within the timeframe analyzed (i.e., last diagnosis in the first year for analyses of the first year of service use, last diagnosis in the second year for analyses of the second year), including AN, BN, OSFED, UFED, and “other” feeding or EDs (pica, rumination disorder, and BED, which were grouped due to low prevalence). Diagnoses were made in a variety of settings by a range of providers (e.g., pediatricians, mental health clinicians). Referrals for follow‐up mental health care may not have always been initiated following diagnosis and there was no one set pathway for referrals when made.

### 1.2 Measures

#### 1.2.1 Service Utilization

Information about service utilization was extracted from claims data, which included the date(s), type, code, and primary and secondary ICD‐9 or ICD‐10 billing diagnoses for each service received. Services were grouped into four categories: outpatient mental health, outpatient medical/physical, inpatient mental health, and inpatient medical/physical. Service use was classified as outpatient if the Medi‐Cal claim type was: (1) outpatient, (2) medical, or (3) early and periodic screening, diagnostic, and treatment/child health and disability prevention, which provides periodic health screenings to Medi‐Cal youth. Service use was classified as inpatient if the claim type was labeled inpatient. Claims were then subcategorized as mental health or medical/physical. For outpatient claims, claims were categorized as mental health if they were a mental health‐related category of service or service type, involved a service with a mental health/substance use provider, or included a primary mental health (including substance use) diagnosis code. Otherwise, they were categorized as medical/physical outpatient. For inpatient claims, the entire care episode was classified as mental health inpatient if ≥ 50% of the claims from admission date to discharge date were mental health‐related; otherwise, it was classified as medical/physical inpatient.

#### 1.2.2 Demographic Variables

Demographic variables included sex, race/ethnicity (collected as a single variable by DHCS with exclusive categories of American Indian/Alaska Native, Asian/Pacific Islander, Black/African American, Hispanic/Latinx, White, and other/unknown), preferred spoken language (English, Spanish, other), and earliest age of known ED diagnosis, which was calculated from birth month and year and the date of the first claims record with an ED diagnosis in the dataset.

### 1.3 Statistical Analyses

#### 1.3.1 Descriptive Analyses

We utilized descriptive statistics along with Chi‐square or ANOVA tests to compare the proportion of youth who received a particular service type in each year and the average number of unique days on which the service type was received in the first and second year following initial diagnosis across ED diagnostic categories. Medians and ranges are reported to offer additional context. The total possible range of days for any service type was 0–365 each year. Additional descriptive analyses across demographic characteristics (age at first diagnosis, sex, race/ethnicity, and preferred language) were conducted to more comprehensively characterize youth who received inpatient services within the first year after known ED diagnosis, who were of special interest due to early utilization of the most intensive services.

#### 1.3.2 Analyses With Covariates

Next, we conducted logistic regressions with both diagnostic and demographic variables included to better understand which factors were uniquely associated with service use in the first year after diagnosis and potential interactions between variables. Models for outpatient services used ordinal logistic regression with odds of greater outpatient mental health and outpatient medical/physical service use as outcomes. Outpatient services were categorized into low, medium, and high utilization, with groupings chosen because they met the proportional odds assumption for ordinal regression. For mental health outpatient services, low use was ≤ 16 days in the year (≤ 4 days per quarter), medium use was 17–36 days (> 4 days per quarter and ≤ 3 days per month), and high use was > 36 days (> 3 days per month). For medical outpatient services, low use was 0–3 days in the year, medium use was 4–5 days, and high use was ≥ 6 days. Models for inpatient services used binary logistic regression with odds of any inpatient mental health admission and any inpatient medical/physical admission as outcomes.

Independent variables were diagnosis, age at first diagnosis, sex, and race/ethnicity. Preferred language was not included in the model due to collinearity with race/ethnicity; however, analyses using preferred language in place of race/ethnicity are included in Table S1. We examined interactions between all independent variables and retained significant interactions in the final models. Analyses were performed in SAS 9.3 and significance was evaluated at *α* = 0.05.

## 2 Results

### 2.1 Service Utilization in the First Year After Known Diagnosis

#### 2.1.1 Descriptive Analyses

##### 2.1.1.1 Outpatient Mental Health

Youth with AN (*M* = 33.6, SD = 37.0) and BN (*M* = 38.2, SD = 45.2) received the most days of outpatient mental health services (all other EDs: *M*s = [20.6, 28.0]; *p*s < 0.01) (see Table 1). When examining specific mental health service codes, participants with AN (35.2%) and BN (44.8%) had the highest rates of family therapy (i.e., sessions with current procedural terminology [CPT] billing codes 90846, 90847, H0032) compared to youth with all other EDs (OSFED: 19.1%, UFED: 29.1%, “other” EDs: 22.4%; *p*s < 0.05). Youth with AN also had the highest rate of individual therapy (CPT codes 90832–90834, 90836–90840, 90875, 90876) relative to youth with all other EDs (20.6%; vs. BN: 13.9%, OSFED: 7.5%, UFED: 15.4%, “other” EDs: 8.7%; *p*s < 0.05). However, the annual number of therapy sessions (4–7 individual therapy sessions and ≈5 family therapy sessions) was remarkably low across diagnoses, without diagnostic differences except that youth with AN (*M* = 6.7, SD = 7.0) and OSFED (*M* = 6.8, SD = 6.6) received slightly more individual therapy than youth with BN (*M* = 4.3, SD = 4.4) (*p*s < 0.05).

**TABLE 1:** Description of service use in the first year after known diagnosis by type of eating disorder.

	AN^a^		BN		OSFED		UFED		Other EDs		All EDs	
First year	*N*	%	*N*	%	*N*	%	*N*	%	*N*	%	*N*	%
All	472	14.3	330	10.0	571	17.2	1616	48.8	322	9.7	3311	100
Sex												
Female	366	77.5	298	90.3	303	53.1	1149	71.1	154	47.8	2270	68.6
Male	106	22.5	32	9.7	268	46.9	467	28.9	168	52.2	1041	31.4
Race												
Asian/Pacific Islander	25	5.3	14	4.2	27	4.7	81	5.0	17	5.3	164	5.0
Black	13	2.8	14	4.2	20	3.5	57	3.5	26	8.1	130	3.9
Latinx	262	55.5	183	55.5	285	49.9	978	60.5	134	41.6	1842	55.6
Other/Unknown	51	10.8	36	10.9	122	21.4	165	10.2	62	19.3	436	13.2
White	121	25.6	83	25.2	117	20.5	335	20.7	83	25.8	739	22.3
Language												
English	243	51.5	165	50.0	288	50.4	781	48.3	223	69.3	1700	51.3
Other	229	48.5	165	50.0	283	49.6	835	51.7	99	30.7	1611	48.7
Age	**Median**	**Mean (SD)**	**Median**	**Mean (SD)**	**Median**	**Mean (SD)**	**Median**	**Mean (SD)**	**Median**	**Mean (SD)**	**Median**	**Mean (SD)**
At known diagnosis	14.3	13.8 (2.4)	15.0	14.6 (1.9)	10.8	11.3 (3.2)	13.8	13.2 (2.7)	9.9	10.7 (2.9)	13.6	12.8 (2.9)
First year services	***N* (%)**	**Mean (SD)**	***N* (%)**	**Mean (SD)**	***N* (%)**	**Mean (SD)**	***N* (%)**	**Mean (SD)**	***N* (%)**	**Mean (SD)**	***N* (%)**	**Mean (SD)**
Outpatient MH days	442 (93.6)	33.6 (37.0)	303 (91.8)	38.2 (45.2)	485 (84.9)	20.6 (31.1)	1387 (85.8)	28.0 (38.6)	291 (90.4)	21.9 (30.7)	2908 (87.8)	28.1 (37.6)
Individual therapy visits	97 (20.6)	6.7 (7.0)	46 (13.9)	4.3 (4.4)	43 (7.5)	6.8 (6.6)	249 (15.4)	5.3 (6.5)	28 (8.7)	5.6 (8.1)	463 (14)	5.7 (6.6)
Family therapy visits	166 (35.2)	5.5 (6.5)	148 (44.8)	5.2 (5.4)	109 (19.1)	4.5 (5.4)	471 (29.1)	4.6 (6.2)	72 (22.4)	4.5 (6.4)	966 (29.2)	4.8 (6.1)
Outpatient MP days	467 (98.9)	13.7 (17.6)	319 (96.7)	10.5 (10.9)	560 (98.1)	30.6 (42.2)	1594 (98.6)	11.5 (15.4)	318 (98.8)	20.7 (24.1)	3258 (98.4)	15.9 (24.2)
Any inpatient admission	131 (27.8)	1.9 (2.0)	99 (30.0)	2.0 (1.6)	105 (18.4)	1.5 (1.1)	260 (16.1)	1.7 (1.2)	27 (8.4)	1.5 (1.4)	622 (18.8)	1.8 (1.5)
Inpatient MH days	17 (3.6)	10.8 (13.5)	10 (3.0)	5.3 (6.8)	68 (11.9)	119.7 (165.8)	39 (2.4)	6.0 (13.0)	17 (5.3)	4.9 (8.2)	492 (14.9)	20.4 (49.2)
Inpatient MP days	119 (25.2)	19.1 (25.6)	92 (27.9)	14.8 (17.9)	43 (7.5)	68.7 (132.6)	228 (14.1)	14.7 (31.8)	10 (3.1)	9.6 (16.6)	151 (4.6)	57.6 (124.6)

*Note*: Individual therapy was identified based on the following Current Procedural Terminology (CPT) codes: 90832–90834, 90836–90840, 90875, and 90876. Family therapy was identified using 90846, 90847, and H0032.

Abbreviations: AN, anorexia nervosa; BN, bulimia nervosa; ED, eating disorder; MH, mental health; MP, medical/physical; OSFED, other specified feeding or eating disorder; SD, standard deviation; UFED, unspecified feeding or eating disorder.

^a^ Reference group.

##### 2.1.1.2 Outpatient Medical

Youth with OSFED (*M* = 30.6, SD = 42.2) had significantly more days of outpatient medical care than youth with other diagnoses (*M*s = [10.5, 20.7]; *p*s < 0.0001). Participants with “other” EDs had a greater number of days of outpatient medical care than participants with AN, BN, or UFED (*p*s < 0.0001).

##### 2.1.1.3 Inpatient Services

Youth with AN (27.8%) and BN (30.0%) had the highest overall hospitalization rates for mental or physical health reasons (OSFED: 18.4%, UFED: 16.1%, “other” EDs: 8.4%; *p*s < 0.01). However, youth with OSFED were most likely to be hospitalized for mental health reasons specifically (11.9%; vs. AN: 3.6%, BN: 3.0%, UFED: 2.4%, “other” EDs: 5.3%; *p*s < 0.01) and had significantly longer mental health admissions than youth with all other diagnoses (*M* = 119.7, SD = 165.8; all other EDs: *M*s = [4.9, 10.8]; *p*s < 0.05). Youth with OSFED also had significantly longer inpatient admissions for primarily medical care than youth with all other diagnoses (*M* = 68.7, SD = 132.6; AN, BN, UFED: *M*s = [14.7, 19.1]; *p*s < 0.01) except “other” EDs (*M* = 9.6), which had low power to detect a significant difference due to the small number of youth in this group experiencing medical hospitalization (*n* = 10).

Youth hospitalized within the first year following diagnosis are further described in Table 2. Across EDs, girls (19.0% vs. boys 6.8%; *p* < 0.0001), English speakers (19.0% vs. other 11.0%; *p* < 0.0001), and White youth (23.0% vs. Black: 15.0%, Latinx: 11.0%, and other/unknown: 15.0%; *p*s < 0.0001) were hospitalized at significantly higher rates for mental health‐focused inpatient care. However, once hospitalized, boys (*M* = 35.5, SD = 83.0) had significantly longer inpatient mental health stays relative to girls (*M* = 17.9, SD = 40.5) (*p* < 0.0001) and youth of other/unknown race had significantly longer stays (*M* = 48.7, SD = 106.5) compared to White, Asian/Pacific Islander, Black, or Latinx youth (*M*s = [8.7, 20.5], *p*s < 0.0001). Although Latinx youth had the lowest rates of mental health‐related hospitalization, their average length of stay was not significantly different than that of White youth.

**TABLE 2:** Demographic factors associated with inpatient service days among youth with EDs in the first year after known diagnosis.

	All	Inpatient mental health days							Inpatient medical/physical days						
	*N*	*N*	%	*p*	Mdn	Mean	Std	*p*	*N*	%	*p*	Mdn	Mean	Std	*p*
All	3311	492	15.0		7.5	20.4	49.2		151	4.6		4	57.6	124.6	
Sex				**				*			ns				ns
Female	2270	421	19.0		7	17.9	40.5		102	4.5		3	50.7	116.3	
Male	1041	71	6.8		10	35.5	83.0		49	4.7		4	71.8	140.7	
Race				**				**			**				**
Asian/Pacific Islander	164	28	17.0	a,b	10	20.5	24.5	b	5	3.0	b,c	12	80.6	159.1	a,b
Black	130	20	15.0	b,c	6.5	8.7	8.0	b	7	5.4	a,b,c	28	114.4	171.7	a,b
Latinx	1842	207	11.0	c	7	14.1	30.0	b	56	3.0	c	3	13.9	49.6	b
Other/Unknown	436	66	15.0	b,c	8	48.7	106.5	a	42	9.6	a	12.5	138.0	172.0	a
White	739	171	23.0	a	10	18.5	32.9	b	41	5.5	b	2	22.3	78.9	b
Language				**				ns			ns				ns
English	1700	321	19.0		7	17.9	40.5		89	5.2		3	66.8	135.5	
Other	1611	171	11.0		8	25.2	62.2		62	3.8		4	44.4	106.9	

*Note*: Values marked with different letters are significantly different from each other.

Abbreviations: Mdn, median; ns, not significantly different; std, standard deviation.

* < 0.01.

** < 0.0001.

With respect to medical/physical hospitalization, youth of other/unknown races had significantly higher rates (9.6%) than White (5.5%), Latinx (3.0%), and Asian/Pacific Islander (3.0%) youth (*p*s < 0.01). Youth of other/unknown race also had significantly longer medical inpatient stays (*M* = 138.0, SD = 172.0) than White and Latinx youth (White: *M* = 22.3, SD = 78.9; Latinx: *M* = 13.9, SD = 49.6; *p*s < 0.0001).

#### 2.1.2 Analyses Including Covariates and Interaction Effects

##### 2.1.2.1 Outpatient Mental Health

**FIGURE 1:**
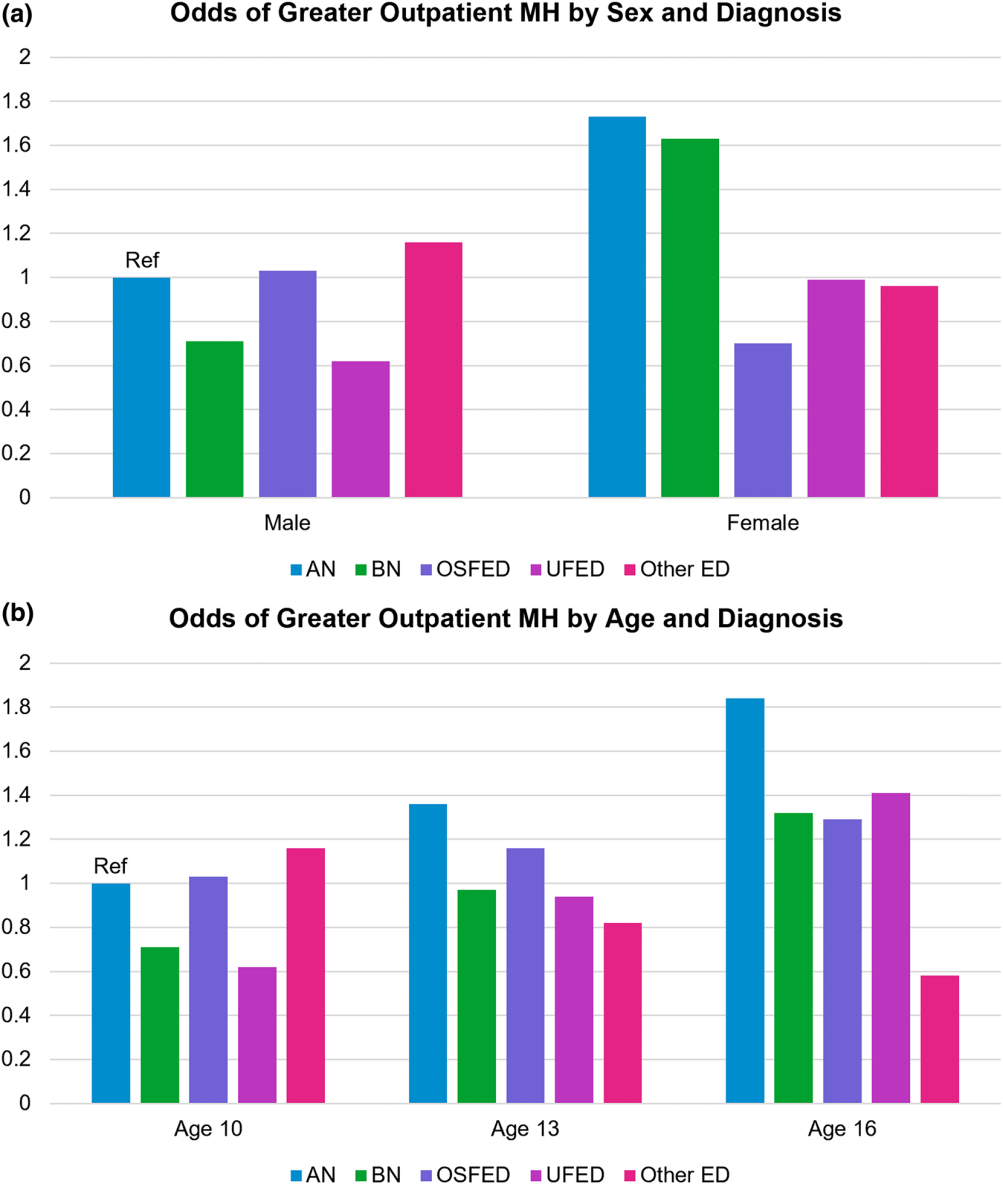
Odds of greater outpatient mental health service use by diagnosis across sex and age in the first year after known diagnosis. AN, anorexia nervosa; BN, bulimia nervosa; ED, eating disorder; MH, mental health service use; OSFED, other specified feeding or eating disorder; UFED, unspecified feeding or eating disorder. The reference group for each graph (male youth with AN, 10‐year‐old youth with AN) is marked with “ref.”

Logistic ordinal regression showed that the relationship between diagnosis and outpatient mental health service use depended on sex and age at first known diagnosis (see Table 3). Differences in outpatient mental health service use between youth with AN/BN and youth with other diagnoses tended to be particularly pronounced for girls (see Figure 1). Girls with AN had significantly higher odds of greater outpatient mental health service use than girls with OSFED (OR = 0.40, 95% CI [0.25, 0.63]), UFED (OR = 0.57, 95% CI [0.38, 0.87]), and “other” EDs (OR = 0.55, 95% CI [0.33, 0.92]), and girls with BN had significantly higher odds than girls with OSFED (OR = 0.43, 95% CI [0.22, 0.83]) (*p*s < 0.05). Within diagnosis, girls were more likely than boys to receive greater outpatient mental health care for AN (OR = 1.73, 95% CI [1.12, 2.69]), BN (OR = 2.29, 95% CI [1.06, 4.98]), and UFED (OR = 1.60, 95% CI [1.27, 2.01]) (*p*s < 0.05).

**TABLE 3:** Interactions between diagnostic and demographic variables in predicting service use in the first year after known diagnosis.

	Greater outpatient mental health	Greater outpatient medical/physical	Any mental health inpatient admission	Any medical/physical inpatient admission
	OR (95% CI)	OR (95% CI)	OR (95% CI)	OR (95% CI)
Diagnostic group (ref = AN)	Interactions with age and sex		Interaction with age	
BN	0.71 (0.28, 1.83)	0.74 (0.56, 0.99)*	0.79 (0.32, 1.99)	0.78 (0.35, 1.73)
OSFED	1.03 (0.62, 1.72)	0.90 (0.69, 1.18)	0.26 (0.13, 0.49)***	3.96 (2.23, 7.04)***
UFED	0.62 (0.38, 1.02)	0.78 (0.63, 0.97)*	0.24 (0.14, 0.44)***	0.70 (0.39, 1.26)
Other EDs	1.16 (0.68, 1.99)	1.08 (0.78, 1.49)	0.14 (0.06, 0.32)***	1.63 (0.80, 3.36)
Age at first known diagnosis	1.11 (1.03, 1.19)**	0.99 (0.96, 1.02)	1.10 (1.00, 1.22)	1.04 (0.98, 1.11)
Sex (ref = male)				
Female	1.73 (1.12, 2.69) *	0.88 (0.74, 1.03)	2.00 (1.51, 2.65)***	1.31 (0.90–1.91)
Race (ref = White)				
Asian/Pacific Islander	0.80 (0.58, 1.12)	0.53 (0.38, 0.75)**	0.70 (0.44, 1.11)	0.51 (0.20, 1.32)
Black	0.60 (0.41, 0.89)*	0.67 (0.46, 0.99)*	0.77 (0.45, 1.31)	0.93 (0.40–2.16)
Latinx	0.61 (0.52, 0.72)***	0.64 (0.54, 0.77)***	0.47 (0.37, 0.59)***	0.56 (0.37–0.85)*
Other/unknown	0.90 (0.71, 1.13)	1.19 (0.91, 1.55)	0.79 (0.56, 1.10)	1.52 (0.96–2.41)
Model interaction terms (if applicable)				
Diagnosis × age				
BN	1.00 (0.87, 1.15)	*—*	1.05 (0.87, 1.26)	*—*
OSFED	0.94 (0.85, 1.03)	*—*	1.11 (0.96, 1.28)	*—*
UFED	1.04 (0.95, 1.13)	*—*	1.21 (1.07, 1.37)**	*—*
Other EDs	0.80 (0.72, 0.90)**	*—*	1.00 (0.79, 1.26)	*—*
Diagnosis × sex				
BN	1.32 (0.54, 3.22)	*—*	*—*	*—*
OSFED	0.39 (0.22, 0.68)**	*—*	*—*	*—*
UFED	0.92 (0.56, 1.51)	*—*	*—*	*—*
Other EDs	0.47 (0.25, 0.90)*	*—*	*—*	*—*

*Note*: Main effects for diagnoses in analyses with a significant diagnosis x age interaction represent odds ratios relative to AN at age 10.

Abbreviations: AN, anorexia nervosa; BN, bulimia nervosa; CI, confidence interval; ED, eating disorder; OR, odds ratio; OSFED, other specified feeding or eating disorder; ref., reference group; UFED, unspecified feeding or eating disorder.

* < 0.05.

** < 0.01.

*** < 0.0001.

While outpatient mental health service use tended to increase with age for youth with most ED diagnoses, the opposite pattern was observed for youth with “other” EDs (see Figure 1). Youth with “other” EDs had similar or higher odds of greater outpatient mental health service use than participants with AN, BN, OSFED, and UFED at age 10. However, by age 16, participants with “other” EDs had significantly lower odds than participants with AN (OR = 0.31, 95% CI [0.15, 0.66]), OSFED (OR = 0.45, 95% CI [0.22, 0.90]), and UFED (OR = 0.41, 95% CI [0.22, 0.78]) (*p*s < 0.05).

Independent of diagnosis, White youth had higher odds of greater outpatient mental health service use than Black/African American (OR = 0.60, 95% CI [0.41, 0.89]) and Latinx youth (OR = 0.61, 95% CI [0.52, 0.72]) (*p*s < 0.05). In supplemental analyses, youth with a preferred language other than English had lower odds of greater mental health service use (OR = 0.82, 95% CI [0.71, 0.94]).

##### 2.1.2.2 Outpatient Medical

Associations between diagnosis and outpatient medical/physical service use did not significantly differ across demographic characteristics. Participants with BN (OR = 0.74, 95% CI [0.56, 0.99]) and UFED (OR = 0.78, 95% CI [0.63, 0.97]) had lower odds of greater medical service use than youth with AN (*p*s < 0.05). White youth had higher odds than youth from all other racial and ethnic groups (ORs = [0.53, 0.67], *p*s < 0.05) except youth of other/unknown races.

##### 2.1.2.3 Inpatient Services

**FIGURE 2:**
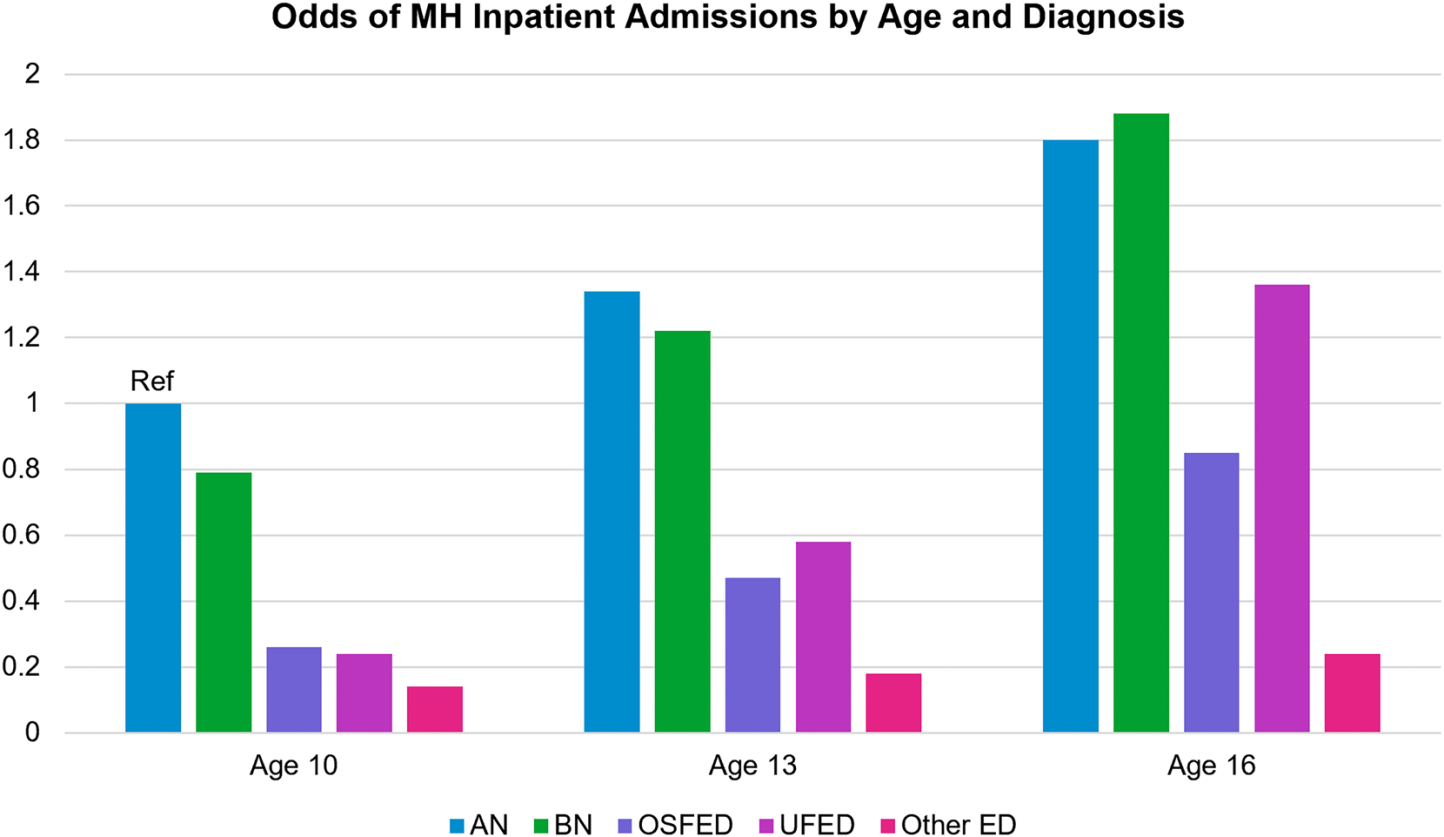
Odds of mental health inpatient admission by diagnosis across age in the first year after known diagnosis. AN, anorexia nervosa; BN, bulimia nervosa; ED, eating disorder; MH, mental health; OSFED, other specified feeding or eating disorder; UFED, unspecified feeding or eating disorder. The reference group (10‐year‐old youth with AN) is marked with “ref.”

For youth with a younger age at first known diagnosis (i.e., at age 10), participants with AN were significantly more likely to experience a mental health inpatient admission than participants with OSFED, UFED, or “other” EDs (ORs = [0.14, 0.26], *p*s < 0.0001). However, this effect was qualified by an age‐by‐diagnosis interaction, such that the difference in odds of mental health hospitalization between AN and UFED decreased with age. At age 16, the odds of mental health hospitalization did not significantly differ between youth with AN and UFED (OR = 0.76, 95% CI [0.54, 1.05]) (see Figure 2). Independent of diagnosis, girls were significantly more likely to experience a mental health hospitalization than boys (OR = 2.00, 95% CI [1.51, 2.65]) and Latinx youth were significantly less likely to experience a mental health hospitalization than White youth (OR = 0.47, 95% CI [0.37, 0.59]). Youth with a preferred language other than English were also less likely to experience a mental health hospitalization in supplemental analyses (OR = 0.50, 95% CI [0.41, 0.62]).

With respect to medical/physical inpatient admissions, odds were greater for youth with OSFED than for youth with all other EDs (ORs = [2.43, 5.63], *p*s < 0.01). Independent of diagnosis, Latinx youth were less likely to experience medical hospitalization compared to White youth (OR = 0.56, 95% CI [0.37, 0.85]; *p* < 0.05).

### 2.2 Descriptive Analyses of Service Use in the Second Year After Known Diagnosis

#### 2.2.1 Outpatient Mental Health

For youth with a second full year of claims after their first known ED diagnosis (*n* = 1293), participants with UFED were least likely to receive any outpatient mental health care (51.3%; vs. AN: 66.9%, BN: 70.8%, OSFED: 66.7%, “other” EDs: 70.1%; *p*s < 0.001) (see Table 4). Rates of individual therapy were low across EDs, but greater for participants with AN (10.1%) and BN (11.5%) relative to participants with OSFED (3.1%), UFED (5.9%), or “other” EDs (4.3%) (*p*s < 0.05). As in the first year after known diagnosis, rates of family therapy were highest for participants with AN (25.3%) and BN (28.1%), and significantly greater among participants with AN compared to participants with UFED (16.0%; *p* < 0.01) and participants with BN relative to participants with OSFED (17.7%; *p* < 0.05), UFED (*p* < 0.01) or “other” EDs (17.1%; *p* < 0.05). The annual number of therapy sessions remained low across diagnoses (~4–7 individual therapy sessions, 3–6 family therapy sessions), with no significant differences by diagnosis except a slightly higher number of family sessions for youth with BN (*M* = 6.3, SD = 7.7) relative to youth with AN (*M* = 3.2, SD = 3.0) (*p* < 0.05).

**TABLE 4:** Description of service use in the second year after known diagnosis by type of eating disorder.

	AN^a^		BN		OSFED		UFED		Other EDs		All EDs	
Second year	*N*	%	*N*	%	*N*	%	*N*	%	*N*	%	*N*	%
All	178	7.7	96	7.4	192	14.8	663	51.3	164	12.7	1293	100
Age	**Median**	**Mean (SD)**	**Median**	**Mean (SD)**	**Median**	**Mean (SD)**	**Median**	**Mean (SD)**	**Median**	**Mean (SD)**	**Median**	**Mean (SD)**
At known diagnosis	13.4	12.7 (2.3)	14.1	13.8 (1.6)	10.0	10.5 (2.6)	13.0	12.3 (2.4)	9.3	10.0 (2.4)	12.5	11.9 (2.6)
Second year services	***N* (%)**	**Mean (SD)**	***N* (%)**	Mean (SD)	***N* (%)**	**Mean (SD)**	***N* (%)**	**Mean (SD)**	***N* (%)**	**Mean (SD)**	***N* (%)**	**Mean (SD)**
Outpatient MH days	119 (66.9)	27.7 (34.9)	68 (70.8)	41.4 (60.5)	128 (66.7)	24.1 (26.9)	340 (51.3)	32.5 (41.8)	115 (70.1)	20.1 (20.4)	770 (59.6)	29.3 (38.6)
Individual therapy visits	18 (10.1)	6.1 (5.4)	11 (11.5)	5.4 (6.0)	6 (3.1)	3.7 (3.1)	39 (5.9)	6.7 (7.3)	7 (4.3)	4.6 (5.6)	81 (6.3)	6 (6.3)
Family therapy visits	45 (25.3)	3.2 (3.0)	27 (28.1)	6.3 (7.7)	34 (17.7)	4.0 (4.0)	106 (16.0)	4.5 (5.9)	28 (17.1)	3.9 (6.4)	240 (18.6)	4.3 (5.6)
Outpatient MP days	171 (96.1)	9.2 (11.4)	95 (99.0)	8.5 (8.0)	186 (96.9)	37.1 (49.2)	639 (96.4)	10.3 (14.8)	161 (98.2)	20 (26.3)	1252 (96.8)	15.2 (26.0)
Any inpatient admission	18 (10.1)	1.6 (1.1)	17 (17.7)	1.7 (1.4)	19 (9.9)	1.6 (1.1)	53 (8.0)	1.5 (1.2)	10 (6.1)	1.4 (1.3)	117 (9.0)	1.5 (1.2)
Inpatient MH days	2 (1.1)	1.5 (0.7)	1 (1.0)	1.0 (0.0)	12 (6.3)	5.6 (5.6)	16 (2.4)	5.1 (6.6)	7 (4.3)	5.9 (7.8)	81 (6.3)	10.7 (13.3)
Inpatient MP days	16 (9.0)	17.6 (19.0)	16 (16.7)	10.8 (10.0)	9 (4.7)	7.0 (3.4)	37 (5.6)	8.7 (13.0)	3 (1.8)	9.7 (6.4)	38 (2.9)	5.1 (6.2)

*Note*: Individual therapy was identified based on the following Current Procedural Terminology (CPT) codes: 90832–90834, 90836–90840, 90875, and 90876. Family therapy was identified using 90846, 90847, and H0032.

Abbreviations: AN, anorexia nervosa; BN, bulimia nervosa; ED, eating disorder; MH, mental health; MP, medical/physical; OSFED, other specified feeding or eating disorder; SD, standard deviation; UFED, unspecified feeding or eating disorder.

^a^ Reference group.

#### 2.2.2 Outpatient Medical

Youth with OSFED (*M* = 37.1, SD = 49.2) continued to have more days of outpatient medical care than youth with all other diagnoses (*M*s = [8.5, 20.0]; *p*s < 0.0001). Participants with “other” EDs in turn had more days of outpatient medical care than participants with AN, BN, or UFED (*p*s < 0.0001).

#### 2.2.3 Inpatient Services

Participants with BN were most likely to experience any inpatient admission during the second year after a known diagnosis (17.7%), and significantly more likely than participants with UFED (8.0%) or “other” EDs (6.1%) (*p*s < 0.01). We did not compare more fine‐grained aspects of inpatient admissions (e.g., number of days) across diagnoses for the second year of claims given smaller sample sizes, but descriptive statistics by diagnosis are reported in Table 4.

## 3 Discussion

This study sought to better understand patterns of service utilization across diagnostic and demographic characteristics in publicly–insured youth with EDs in the US. Across diagnoses, rates, and frequency of individual and family therapy were low, while rates of hospitalization were high. Nevertheless, there were also some important differences in service use by diagnosis. For example, youth with OSFED appeared to have particularly high physical health needs, as they were almost four times as likely to experience a medical inpatient admission than youth with AN. Conversely, youth with EDs other than AN and BN had fewer days of outpatient mental health treatment and were less likely to receive family and (relative to AN) individual therapy, with differences in outpatient mental health treatment particularly pronounced for girls. We also found significant treatment disparities across race/ethnicity, sex, and preferred language. Overall, these results significantly extend our understanding of the treatment experiences of publicly–insured youth with EDs and highlight potential disparities across diagnosis and demographic factors in this population.

Notably, a substantial proportion of youth received no outpatient individual or family therapy at all in the first year after known ED diagnosis. Among youth who did receive therapy, the annual number of sessions was far below that recommended in evidence‐based protocols (e.g., family‐based treatment [FBT], enhanced cognitive behavioral therapy [CBT‐E]; Atwood and Friedman 2020; Gorrell, Loeb, and Le Grange 2019; Le Grange et al. 2022) and less than half that observed for adolescents with EDs in other naturalistic settings (Lindstedt et al. 2020; Oshukova et al. 2023; Simic et al. 2022). Youth with Medi‐Cal may face multiple barriers to outpatient ED treatment (Crest et al. 2024), including a dearth of ED‐trained Medi‐Cal providers (Accurso, Buckelew, and Snowden 2021) and logistical obstacles (e.g., transportation, ability for parents to take time off work). Conversely, hospitalization rates were high, particularly for youth with AN (28%) and BN (30%). Hospitalization rates were greater than in other studies of youth with EDs (e.g., 5.4% among youth with private Kaiser insurance; Lau et al. 2022), which could reflect limited access to appropriate outpatient care and/or delayed recognition leading to increased severity at the time of diagnosis. Evidence‐based outpatient treatment for EDs costs substantially less than inpatient care (Streatfeild et al. 2021) and reduces the likelihood of hospitalization in adolescents (Lock et al. 2016). Additional research is needed to explore whether increasing access to outpatient therapy could reduce the need for hospitalization among publicly–insured youth.

Medical acuity appeared particularly high for youth with OSFED, who had substantially higher odds of hospitalization for primarily medical reasons and significantly more days of outpatient medical care than youth with other diagnoses. Although youth with OSFED received fewer days of outpatient mental health services than participants with AN and BN (particularly among girls), when hospitalized for mental health reasons, they had substantially longer stays than youth with other diagnoses (*M* = 120 days). Treatment needs may go underrecognized among youth with OSFED until their disorder is severe, contributing to elevated acuity at the time of diagnosis and lengthy inpatient stays. Results add to evidence that OSFED is no less serious than AN/BN in adolescents (Ernst, Bürger, and Hammerle 2017; Fairweather‐Schmidt and Wade 2014) and may be associated with increased barriers to appropriate treatment (Penwell et al. 2024).

A question of interest in the current study was whether having an unspecified diagnosis might be associated with treatment disparities. Like youth with OSFED, those with UFED received fewer days of outpatient mental health services and were less likely to receive family or individual therapy than youth with AN. Unlike youth with OSFED, however, youth with UFED did not receive higher‐intensity medical care. Youth with UFED were also less likely to experience a mental health‐related hospitalization than youth with AN, particularly at younger ages. Disparities between youth with UFED and those with other diagnoses were more pronounced in the second year after a known diagnosis when participants with UFED had the lowest rates of any outpatient mental health care and individual or family therapy. While one possible explanation could be that youth with UFED have less severe presentations, existing research suggests symptom severity may not significantly differ in UFED relative to specified/threshold EDs in adolescents (Wade and O'Shea 2015). Alternatively, youth with UFED may be less likely to receive follow‐up care despite having similar needs to youth with other EDs (Moreno et al. 2023). UFED is intended to be used when there is insufficient time/information to make a more specific diagnosis (e.g., emergency room settings; American Psychiatric Association 2022), and youth evaluated under such circumstances may be more easily “lost in the shuffle” in systems of care. Further research is needed to better understand the experiences of publicly–insured youth with UFED (who were a plurality of the population) and the extent to which they have unmet needs.

Latinx and Black/African American youth received significantly less outpatient care than their White counterparts after accounting for diagnosis, and Latinx youth were also less likely to be hospitalized. Youth with a preferred language other than English were less likely to be hospitalized for mental health reasons but were equally likely to be hospitalized for physical health reasons and had equivalent lengths of stay once admitted, suggesting similar severity. These results are particularly striking given that nearly 60% of youth were Latinx and approximately half had a preferred language other than English. Findings are consistent with research suggesting Latinx youth and youth with a preferred language other than English are less likely to receive recommended ED treatment (Moreno et al. 2023). Black and Latinx youth who do not fit ED stereotypes may be less likely to have their ED severity recognized and be referred for specialized/intensive care (Mikhail and Klump 2021). Linguistic and cultural barriers (Acle et al. 2021), stigma (Neyland and Bardone‐Cone 2019), and fear of or experienced bias from providers (Reyes‐Rodríguez et al. 2013) may also impede access to and retention in treatment for minoritized youth and families. Similarly, boys tended to receive less outpatient mental health treatment than girls (particularly for AN and BN). Boys were also less likely to be admitted for a mental health‐related hospitalization but remained in the hospital longer once admitted, suggesting equal or greater needs. Boys may find it more difficult to engage in therapy designed primarily with female populations (Sangha et al. 2019) or their needs may go underrecognized until their symptoms are relatively severe, necessitating longer inpatient stays.

This study has several strengths, including a large, comprehensive sample of publicly–insured youth with EDs and diversity across diagnosis, sex, language, and race/ethnicity. Nevertheless, there are some limitations. Youth were exclusively drawn from California and results may not generalize to other states with different treatment contexts and demographic characteristics. Race and ethnicity are collected by DHCS as a single exclusive variable; thus, we were not able to analyze nuances of treatment experiences for youth with multiple racial/ethnic identities. Age at known diagnosis was based on the first claim with a documented ED diagnosis in the dataset, which could be later than the actual age of onset for youth whose ED onset before the sampling period. We did not have data on gender identity to examine potential disparities in treatment among gender‐diverse youth. Diagnoses were assigned by a variety of providers across diverse contexts; limited time or competing priorities within the health encounter and/or limited provider experience with EDs may have contributed to high rates of UFED. Relatedly, there were no youth with diagnosed avoidant/restrictive food intake disorder (ARFID) in the population (Accurso et al. 2024), with these youth likely falling in the OSFED or UFED categories. Billing claims do not provide details regarding symptom profiles; thus, we could not examine specific OSFED/UFED presentations (e.g., purging disorder, atypical AN). Moreover, there were likely many youth with EDs who never received a diagnosis and thus were not captured in this study.

Future research should investigate the origins of disparities and whether unique factors contribute to disparities for youth with different identities (e.g., whether Black and Latinx youth experience different barriers to care). Research on more recent experiences among Medi‐Cal youth would also be informative, particularly following the general increase in hospitalization for youth EDs during the COVID‐19 pandemic (Milliren, Richmond, and Hudgins 2023). More detailed information is also needed regarding the specific treatments delivered and whether these align with evidence‐based guidelines (e.g., the extent to which FBT was delivered in the context of family therapy claims). Finally, qualitative research can provide unique insight into what families with Medi‐Cal view as helpful about current treatment and where they perceive gaps in care.

## Author Contributions

**Megan E. Mikhail:** conceptualization, formal analysis, visualization, writing – original draft. **Kate Duggento Cordell:** formal analysis, investigation, methodology, software, writing – review and editing. **Amanda E. Downey:** conceptualization, investigation, methodology, writing – review and editing. **Lonnie R. Snowden:** conceptualization, data curation, investigation, methodology, writing – review and editing. **Erin C. Accurso:** conceptualization, data curation, funding acquisition, investigation, methodology, supervision, writing – review and editing.

## Ethics Statement

Study procedures were approved by the University of California, San Francisco, and California Committee for the Protection of Human Subjects Institutional Review Boards.

## Conflicts of Interest

Dr. Accurso has consulted with Partnership HealthPlan of California (a healthcare organization that contracts with the state to administer Medicaid benefits) concerning strategies to improve the treatment of EDs. The other authors have no conflicts to declare.

## Supporting information


Table S1.

## Data Availability

The data that support the findings of this study are available directly from the California Department of Health Care Services.
