# Supplementary material for: Predictors of Outpatient and Inpatient Service Utilization Among Publicly–Insured Youth With Eating Disorders
Source: Int J Eat Disord. 2024 Oct 24;58(1):181–92. doi: 10.1002/eat.24301 (PMC11784826; doi:10.1002/eat.24301)
Supplement: Supplementary file 1 — Table S1. [file EAT-58-181-s001.docx]

**Supplemental Material**

**Table S1. Interactions between Diagnostic and Demographic Variables in Predicting Service Use in First Year after Known Diagnosis, with Preferred Language as a Predictor Instead of Race/Ethnicity**

|  |  | **Greater Outpatient**  **Mental Health** | **Greater Outpatient Medical/Physical** | **Any Mental Health Inpatient Admission** | | **Any Medical/Physical Inpatient Admission** | |
| --- | --- | --- | --- | --- | --- | --- | --- |
|  |  | OR (95% CI) | OR (95% CI) | OR (95% CI) | | OR (95% CI) | |
|  |  |  |  |  | |  |  |
| **Diagnostic Group (Ref = AN)** | | ***Interactions with age and sex*** |  | ***Interaction with age*** | |  | |
|  | |  |  |  | |  | |
| BN | | .71 (.28, 1.83) | .75 (.56, 1.00) | .89 (.35, 2.25) | | .79 (.36, 1.75) | |
| OSFED | | 1.09 (.65, 1.80) | .95 (.72, 1.23) | **.28 (.15, .54) **** | | **4.25 (2.41, 7.50) ***** | |
| UFED | | .59 (.36, .98) * | **.77 (.62, .95) *** | **.25 (.14, .45) ***** | | .69 (.39, 1.24) | |
| Other EDs | | 1.19 (.70, 2.04) | 1.13 (.82, 1.55) | **.14 (.06, .33) ***** | | 1.72 (.84, 3.53) | |
|  | |  |  |  | |  | |
| **Age at First Known Diagnosis** | | **1.12 (1.04, 1.21) **** | .99 (.97, 1.02) | **1.13 (1.02, 1.24) *** | | 1.04 (.98, 1.11) | |
|  | |  |  |  | |  |  |
| **Sex (Ref = Male)** | |  |  |  | |  |  |
|  | Female | **1.65 (1.06, 2.55) *** | .87 (.74, 1.02) | **1.96 (1.48, 2.59)** *** | | 1.24 (.86, 1.80) | |
|  | |  |  |  | |  |  |
| **Language (Ref = English)** | |  |  |  | |  |  |
| Other Language | | **.82 (.71, .94) **** | .91 (.79, 1.05) | **.50 (.41, .62) ***** | | .75 (.54, 1.06) | |
|  | |  |  |  |  |  |  |
| **Model Interaction Terms** | |  |  |  |  |  |  |
| *(if applicable)* | |  |  |  |  |  |  |
|  |  |  |  |  |  |  |  |
| **Diagnosis x Age** | |  |  |  |  |  |  |
| BN | | .99 (.86, 1.14) | ***—*** | 1.02 (.85, 1.23) | | ***—*** | |
| OSFED | | .92 (.84, 1.01) | ***—*** | 1.08 (.94, 1.25) | | ***—*** | |
| UFED | | 1.03 (.95, 1.12) | ***—*** | **1.19 (1.06, 1.35) **** | | ***—*** | |
| Other EDs | | **.80 (.71, .89) ***** | ***—*** | .98 (.77, 1.23) | | ***—*** | |
|  | |  |  |  |  |  |  |
| **Diagnosis x Sex** | |  |  |  |  |  |  |
| BN | | 1.39 (.57, 3.39) | ***—*** | ***—*** | | ***—*** | |
| OSFED | | **.40 (.23, .70) **** | ***—*** | ***—*** | | ***—*** | |
| UFED | | .99 (.60, 1.62) | ***—*** | ***—*** | | ***—*** | |
| Other EDs | | **.49 (.26, .92) *** | ***—*** | ***—*** | | ***—*** | |

** <0.05, ** <0.01, *** <0.0001*

***Note*.** AN = anorexia nervosa; BN = bulimia nervosa; OSFED = other specified feeding or eating disorder; UFED = unspecified feeding or eating disorder; ED = eating disorder; ref = reference group; OR = odds ratio; CI = confidence interval. Main effects for diagnoses in analyses with a significant diagnosis x age interaction represent odds ratios relative to AN at age 10.
